# Multidisciplinary Views on Applying Explicit and Implicit Motor Learning in Practice: An International Survey

**DOI:** 10.1371/journal.pone.0135522

**Published:** 2015-08-21

**Authors:** Melanie Kleynen, Susy M. Braun, Sascha M. C. Rasquin, Michel H. C. Bleijlevens, Monique A. S. Lexis, Jos Halfens, Mark R. Wilson, Rich S. W. Masters, Anna J. Beurskens

**Affiliations:** 1 Research Centre for Autonomy and Participation of Persons with a Chronic Illness, Faculty of Health, Zuyd University of Applied Sciences, Heerlen, The Netherlands; 2 Adelante Rehabilitation Centre, Department of Brain Injury, Hoensbroek, The Netherlands; 3 Department of Family Medicine, CAPHRI, School for Public Health and Primary Care, Faculty of Health, Medicine and Life Sciences, Maastricht University, Maastricht, The Netherlands; 4 Department of Rehabilitation Medicine, CAPHRI, School for Public Health and Primary Care, Faculty of Health, Medicine and Life Sciences, Maastricht University, Maastricht, The Netherlands; 5 Department of Health Services Research, CAPHRI, School for Public Health and Primary Care, Faculty of Health, Medicine and Life Sciences, Maastricht University, Maastricht, The Netherlands; 6 Adelante Centre of Expertise in Rehabilitation and Audiology, Hoensbroek, The Netherlands; 7 Innovation Platform Sevagram, Sevagram Zorgcentra, Heerlen, the Netherlands; 8 Research Centre for Technology in Care, Zuyd University of Applied Sciences, Heerlen, The Netherlands; 9 Department of Sport and Health Sciences, University of Exeter, Exeter, United Kingdom; 10 Institute of Human Performance, University of Hong Kong, Hong Kong, China; 11 Te Oranga School of Human Development and Movement Studies, University of Waikato, Hamilton, New Zealand; University of Catania, ITALY

## Abstract

**Background:**

A variety of options and techniques for causing implicit and explicit motor learning have been described in the literature. The aim of the current paper was to provide clearer guidance for practitioners on how to apply motor learning in practice by exploring experts’ opinions and experiences, using the distinction between implicit and explicit motor learning as a conceptual departure point.

**Methods:**

A survey was designed to collect and aggregate informed opinions and experiences from 40 international respondents who had demonstrable expertise related to motor learning in practice and/or research. The survey was administered through an online survey tool and addressed potential options and learning strategies for applying implicit and explicit motor learning. Responses were analysed in terms of consensus (≥ 70%) and trends (≥ 50%). A summary figure was developed to illustrate a taxonomy of the different learning strategies and options indicated by the experts in the survey.

**Results:**

Answers of experts were widely distributed. No consensus was found regarding the application of implicit and explicit motor learning. Some trends were identified: Explicit motor learning can be promoted by using instructions and various types of feedback, but when promoting implicit motor learning, instructions and feedback should be restricted. Further, for implicit motor learning, an external focus of attention should be considered, as well as practicing the entire skill. Experts agreed on three factors that influence motor learning choices: the learner’s abilities, the type of task, and the stage of motor learning (94.5%; n = 34/36). Most experts agreed with the summary figure (64.7%; n = 22/34).

**Conclusion:**

The results provide an overview of possible ways to cause implicit or explicit motor learning, signposting examples from practice and factors that influence day-to-day motor learning decisions.

## Introduction

The acquisition and improvement of motor skills is important to a range of people in different target populations, including athletes and patients in rehabilitation. Although these populations may not at first seem to be comparable, both seek to develop motor performance that is beyond their current capabilities in terms of temporal, spatial and environmental demands of the task [1,2]. Both coaches and therapists must decide how to shape motor performance by selecting from a variety of possible motor learning solutions.

There is a growing body of scientific evidence that reports on approaches designed to influence motor learning [3]. However, the circumstances in which motor learning approaches are applied during practice and research are often different. Researchers mainly apply motor learning interventions in controlled circumstances according to a protocol, often within a highly selected population. Practitioners (e.g., physical therapists), on the other hand, mainly apply motor learning in circumstances that are less controllable and more variable. To aggregate and compare knowledge and opinions from different fields, an international survey of researchers and professionals with different backgrounds and demonstrable experience of motor learning in sports, rehabilitation and research was performed [4]. The distinction between implicit and explicit motor learning was used as a conceptual departure point. The distinction between these two forms of motor learning has often been used in laboratory (e.g., [5–7]) and clinical research (e.g., [8–10]), as well as in overview papers [11–15], in a variety of fields and for different skills.

We have previously reported our results from earlier rounds of the survey, which focused on definition and classification issues related to implicit and explicit motor learning [16]. Consensus was reached on the definitions of both explicit and implicit motor learning. Explicit motor learning was defined by experts as “learning which generates verbal knowledge of movement performance (e.g., facts and rules), involves cognitive stages within the learning process and is dependent on working memory involvement”, whereas implicit learning was defined as “learning which progresses with no or minimal increase in verbal knowledge of movement performance (e.g., facts and rules) and without awareness”. The experts further identified seven common motor learning intervention strategies (in this article referred to as best-known strategies): discovery learning, analogy learning, errorless learning, observational learning, dual task learning, trial and error learning, and movement imagery [16].

Although there seems to be agreement on the definitions of terms related to motor learning, there are lot of examples of different methods and techniques used to apply implicit or explicit motor learning. For example, Masters “caused” implicit motor learning by asking participants to carry out a concurrent random letter generation task while practicing a golf-putting task (dual-task) or by using a biomechanical metaphor (analogy) [17,18]. More recently, McCombe Waller and Prettyman probably caused implicit learning of a balancing task by asking participants to focus on a different task (i.e., grasp, release and reach) during upright standing [19] and Van Tilborg et al. used modeling (observation) to facilitate implicit motor learning of every day instrumental ADL (activities of daily living) tasks [8]. In other work, the environment has been manipulated during learning of tasks, such as dynamic balancing or throwing [9, 20], in order to promote or reduce errors, which is thought to facilitate explicit or implicit motor learning respectively. Typically, explicit learning has been operationalized by instructing learners to discover rules by themselves [20] or by providing them with verbal instructions or rules [8,10].

The aim of the current paper was to provide clearer guidance for practitioners on how to influence motor learning in practice. This was achieved by exploring experts’ opinions and experiences of how motor learning can be applied in practice, using the distinction between implicit and explicit motor learning as a conceptual departure point.

## Method

### Design

This study was part of a larger Delphi survey [4]. Three sequential survey rounds, interspersed by controlled feedback, were used to collect and aggregate informed judgements from a group of experts on different aspects of motor learning. The study was designed and distributed using an online survey programme (SurveyMonkey, SurveyMonkey.com, LLC, California, USA).

In this article, we present results of the second and the third round, which addressed the application of motor learning approaches in practice. More detailed information about the method and rationale for the entire survey is presented elsewhere [4]. The Central Ethics Committee Atrium-Orbis-Zuyd (Institutional Review Board, IRB) was contacted and formal written permission to perform the study described in the protocol [4] was obtained (13-N-144). According to the IRB, this study was exempt from IRB review, based on the law Medically Scientific Research with people, because there is no way of submitting people to actions or to impose upon them certain behaviors.

A referee group (all authors of the paper) consisting of seven researchers with backgrounds in epidemiology, physiotherapy, occupational therapy, movement sciences and psychology, all with experience of working with different target groups (e.g., sports, general population, rehabilitation, geriatric care), supervised and monitored the process. Two members of the referee group (MK, SB) were responsible for distributing and monitoring the survey (e.g., sending reminders and feedback reports).

### Study population

A panel of international experts was invited to participate in the survey. Members of the panel were initially identified via a literature search and/or the networks of the referee group S1 Fig. Criteria for selection of an expert were based on either scientific publication(s) in the field of motor learning (researcher) or at least three years of working experience applying motor learning in practice plus involvement in education or research (therapist, coach, lecturer) [4]. Experts gave informed consent to participate in the study within the online survey programme.

### Content of the survey

First, experts were asked to state 1) how they would apply implicit and explicit motor learning and 2) how they would apply the seven best-known motor learning strategies [16]. A list of options (in this paper called “elements”) that could be used to operationalize motor learning in practice was provided. Elements included the use of instructions, focus of attention, manual guidance, environmental constraints, variation and feedback and were based on the results of a general literature review, which was conducted when preparing the survey [4]. Experts were asked how they would cause a more implicit or explicit form of learning by using these elements (Round 2). To make answers comparable, primarily predefined categories of response options were used; however, experts had the opportunity to comment on each question (either by using the option “I cannot state as it depends on…” or by using an ‘open text box’) S1 Table.

**Fig 1:**
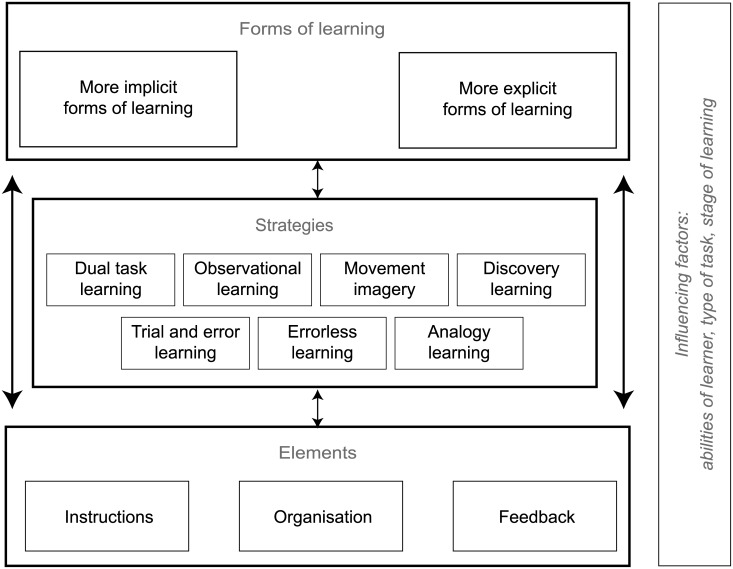
First version summary figure.

During interim analysis, it was decided that it would be appropriate to try to synthesise the large amount of information provided by the experts into a more comprehensible overview using a summary figure (Fig 1). The aim of this summary figure was to present the different strategies and elements suggested in the survey together with a possible taxonomy. The elements were therefore clustered into three categories: instructions (instructions on task, instruction on focus of attention, and manual guidance), feedback (content and timing of the feedback) and organisation (environmental constraints, amount of variation, and division of the skill).

In the subsequent round (Round 3), the experts were asked to state whether they 1) agreed with the figure, 2) agreed with the figure subject to modifications (an open comment box was provided for suggested modifications) or 3) did not agree with the figure.

### Data Analysis

Data analysis was conducted blind to the names and characteristics of the expert respondents. Responses to multiple-choice questions were presented as percentages or absolute numbers. Additionally, the level of agreement with regard to operationalization of motor learning was explored. According to earlier rounds of the survey, consensus on a topic was reached if ≥ 70% of the experts agreed on one option of a multiple-choice question [4, 21–23]. As the study had an exploratory character, consensus was not expected for all questions. Therefore, answers were also analysed using trends (≥50%).

Ninety-five percent confidence intervals for proportions are presented if possible using the Clopper-Pearson exact method [24]. Answers to questions for which experts could choose multiple Es are presented using absolute numbers, together with the percentages (and 95% CI’s) of experts who chose identical combinations of multiple answer options. Subgroup analysis was performed to check whether there was a difference between the answers of experts with a more theoretical/scientist/management role and of experts with a more practical role (e.g., therapists and lecturer). This categorisation was based on the answer to the question “within the past 5 years I mainly worked as” (Table 1). Fisher’s exact test was used and significance criteria was set at α = .05.

**Table 1:** Characteristics of the participating experts.

Category	Subcategory	Results (absolute numbers)
Gender (n = 40)	Male	19
	Female	21
Age category (n = 39)	20–30:	1
	30–40:	11
	40–50:	16
	50–60:	8
	60–70:	-
	> 70:	2
	Not wanted to state/missing:	2
Working country (n = 39)	England/UK:	14
	The Netherlands:	7
	USA:	4
	Australia:	2
	Canada:	3
	France:	2
	Belgium:	-
	Germany:	2
	China/Hong Kong:	3
	New Zealand:	1
	Switzerland:	1
	Missing:	1
In last 5 years mainly worked as (n = 39)	Researcher:	21
	Lecturer/Educator:	5
	Therapist:	8
	Other (e.g., consultant, psychologist):	5
	Missing:	1
Background* (n = 40)	Rehabilitation Practitioner (PT, OT, ST^#^):	19
	Movement Scientist:	14
	Psychologist:	9
	Coach:	6
	Other (e.g. sport scientist, podiatrist):	4
Expert in which motor learning area* (n = 40)	Rehabilitation:	28
	Sports:	15
	Fundamental research (neuroscience):	13
	Elderly:	7
	Children:	2
	Education:	1
	Other (e.g., cognitive psychology, mental health):	3
Target population working with* (n = 40)	Neurological patients (adults):	19
	Elderly:	12
	Healthy population in general:	12
	Athletes:	8
	Neurological patients (children):	4
	Orthopaedic patients (adults):	1
	Healthy children:	1
	Not a practitioner:	3
Years of experiences (n = 40)	Research	Mean: 12.4 (SD: 11.9)
	Practice	Mean: 10.9 (SD:9.5)

*multiple answer options were possible.

^#^PT: Physiotherapist, OT: Occupational Therapist, ST: Speech and Language Therapist.

Free text comments and answers from open questions were described and, if possible, categorised. For example, experts were asked to state whether they would provide instructions when applying implicit or explicit learning in an open comment box and their **r**esponses were categorised into: “Yes (no further explanation)”,”Yes with further explanation”, “No (no further explanation)”,”No further explanation” and “other”. These answers were presented in a figure together with additional subcategories.

## Results

Forty-four experts agreed to participate in the portion of the survey that is presented here. Four experts were excluded from analysis as they skipped all questions in this part of the survey. Two of these experts had mainly worked as researchers during the last five years, one mainly as lecturer and one as a therapist. One of the excluded experts indicated in earlier parts of the survey that he/she did not agree with the design and content of the overall survey. Characteristics of the remaining 40 experts are shown in Table 1. The population of experts was heterogeneous with regard to age, background and current working situation. Not all experts responded to every question. In the text below the number of experts reflects the actual number of participants who answered a certain question.

### Facilitation of implicit and explicit motor learning

**Fig 2:**
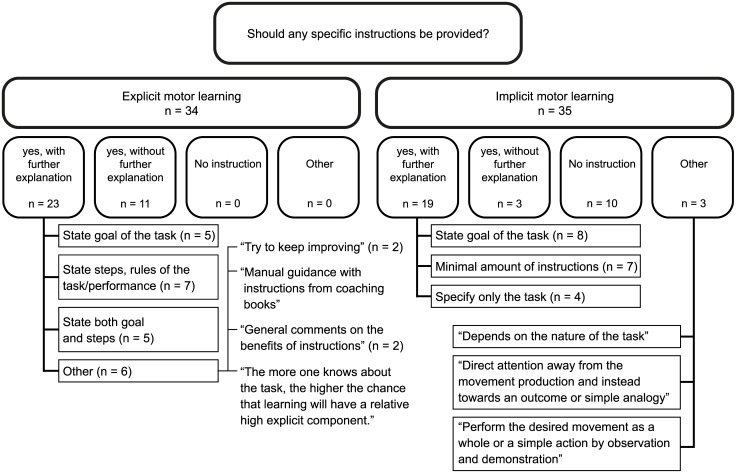
Overview of answers regarding instructions to promote implicit or explicit motor learning.

Most experts stated that, to promote explicit learning, instructions should be provided (n = 29/34) and should either include ‘the goal of the task’, ‘the steps or rules that need to be followed’, or a combination of both. Most experts stated that, to promote implicit learning, instructions should be limited or avoided (n = 25/35). The detailed answers to the open ended questions regarding the use of instructions in explicit and implicit motor learning are illustrated in Fig 2.

The responses with regard to focus of attention, manual guidance, environmental constraints, variation and division of the skill were widely distributed (Table 2). Two trends were found: when applying implicit motor learning, 50% of the experts (n = 20/40) stated that they would choose an external focus of attention and 52.5% of the experts mentioned that the entire skill should be practiced (n = 21/40). Subgroup analysis was performed and revealed no significant difference between the two groups for either of the elements presented in Table 2 (all p-values>.05).

**Table 2:** Overview answers regarding the use of focus of attention, manual guidance, environmental constraints, variation and the division of the skill into parts in implicit and explicit motor learning.

Elements	Answer options	Implicit	95% CI	Explicit	95% CI
Focus of attention (n = 40)	Internal:	10%	2.8%-23.7%	32.5%	18.6%-49.1%
	External:	**50%** *	33.8%-66.2%	32.5%	18.6%-49.1%
	Not applicable:	22.5%	10.8%-38.5%	2.5%	0.0%-13.2%
	Depends on:	17.5%	7.3%-32.8%	32.5%	18.6%-49.1%
Manual Guidance (n = 40)	Much:	2.5%	0.0%-13.2%	10%	2.8%-23.7%
	Some:	10%	2.8%-23.7%	30%	16.6%-46.5%
	Little:	17.5%	7.3%-32.8%	12.5%	4.2%-26.8%
	No:	17.5%	7.3%-32.8%	2.5%	0.0%-13.2%
	Not applicable:	12.5%	4.2%-26.8%	10%	2.8%-23.7%
	Depends on:	40%	24.9%-56.7%	35%	20.6%-51.7%
Environmental constraints (n = 40)	Highly constrained:	15%	5.7%-29.8%	7.5%	0.2%-20.4%
	Somewhat constrained:	25%	12.7%-31.2%	35%	20.6%-51.7%
	Few constraints:	25%	12.7%-31.2%	17.5%	7.3%-32.8%
	No constraints:	7.5%	0.2%-20.4%	10%	2.8%-23.7%
	Not applicable:	-	-	5%	0.6%-16.9%
	Depends on:	27.5%	14.6%-43.9%	25%	12.7%-31.2%
Variation (n = 40)	Much:	22.5%	10.8%-38.5%	32.5%	18.6%-49.1%
	Some:	22.5%	10.8%-38.5%	17.5%	7.3%-32.8%
	Little:	17.5%	7.3%-32.8%	22.5%	10.8%-38.5%
	No:	2.5%	0.0%-13.2%	-	-
	Not applicable:	-	-	-	-
	Depends on:	35%	20.6%-51.7%	27.5%	14.6%-43.9%
Division of skill into parts (n = 40)	Divide into parts:	5%	0.6%-16.9%	45%	29.3%-61.5%
	Practice entire skill:	**52.5%**	36.1%-68.5%	12.5%	4.2%-26.8%
	Not applicable:	12.5%	4.2%-26.8%	5%	0.6%-16.9%
	Depends on:	30%	16.6%-46.5%	37.5%	22.7%-54.2%

*Answers for which ≥ 50% of the experts agreed are bold (trend).

Experts could choose four options regarding the content and the timing of feedback when promoting implicit or explicit motor learning. For explicit learning, 60% (n = 24/40; 95%CI: 43.3%-75.1%) of the experts chose all four feedback content options (performance, results, good and improvable aspects), whereas for implicit motor learning, 42.5% (n = 17/40; 95% CI: 27.0%-59.1%) of the experts only chose feedback of results and 25% (n = 10/40; 95% CI: 13.3%-41.2%) chose none of the content options, selecting only options “not applicable” or “depends on”. With regard to the timing of feedback in explicit learning, answers by the experts group were broadly distributed. A similar trend was evident for the timing of feedback when promoting implicit learning, with 40% (n = 16/40; 95% CI 24.7%-56.7%) of the experts choosing “not applicable” or “depends on”, rather than one of the available timing options. Again, subgroup analysis revealed no significant difference between the two groups for choices regarding the content and timing of feedback (all p-values>.05).

Cumulative responses by all experts regarding the use of feedback when promoting implicit or explicit motor learning are shown in Table 3.

**Table 3:** Frequency count of all selected options regarding content and timing of feedback.

	Answer options	Implicit	Explicit
Feedback (content) (n = 40)	Feedback about the performance:	8	36
	Feedback about the results:	26	32
	Addressing good aspects:	9	33
	Addressing aspects which should be improved:	5	32
	Not applicable:	5	-
	Depends on:	8	2
Feedback (timing) (n = 40)	During movement:	2	19
	After movement:	10	17
	Immediately after action:	6	22
	Delayed:	13	12
	Not applicable:	10	2
	Depends on:	10	12

### Facilitation of the seven best-known motor learning strategies

Of the experts, 72.5% (n = 29/40) agreed that the motor learning elements provided (e.g., instructions, focus of attention) were specific to the seven best-known learning strategies. Some trends could be identified; however, given that on average n = 22 experts skipped questions in this part of the questionnaire (min: n = 16, max: n = 28), data were not representative and are therefore not presented. Experts did not provide reasons for skipping this part of the questionnaire.

### Factors influencing motor learning choices

Within the survey, for the multiple-choice questions, the option ‘I cannot state, since it depends on…’ was chosen frequently (on average 11 of the 40 experts chose that option (27.5%)) (see Table 2). Many experts used the opportunity to clarify their responses and consistently suggested three factors that influence the choice of a specific motor learning intervention: type of task/skill, the learner’s ability (physical, cognitive) and the stage of learning. Subsequently, these three factors were presented for verification in the next survey round.

Most of the experts, 94.5% (n = 33/34; 95% CI: 84.7–99.9%) agreed that these are the most important factors upon which to base motor learning content. Just under half of the experts, 47.1% (n = 16/34; 95% CI: 29.8%-64.9%), reported that the learner’s ability is the most influential factor when selecting motor learning content, and 42.2% (n = 14/34; 95% CI: 24.7%-59.3%) of the experts reported that the type of task or skill to be learned is the most influential factor (e.g., complexity of the task). Only 11.8% (n = 4/34; 95% CI: 3.3%-27.5%) of the experts reported that the stage of motor learning is most influential (e.g., initial or late stage).

Eight experts (5 researchers, 1 lecturer, 1 therapist, 1 manager) failed to complete this part of the survey. Six experts did not provide a reason for dropping out. One researcher dropped out because he/she did not agree with the content and set-up of the survey and one manager stated that he/she could not respond due to lack of time. Two experts (both lecturers) only participated in this second part (Round 3) of the survey after they had failed to complete the earlier part (Round 2).

### Summary figure

**Fig 3:**
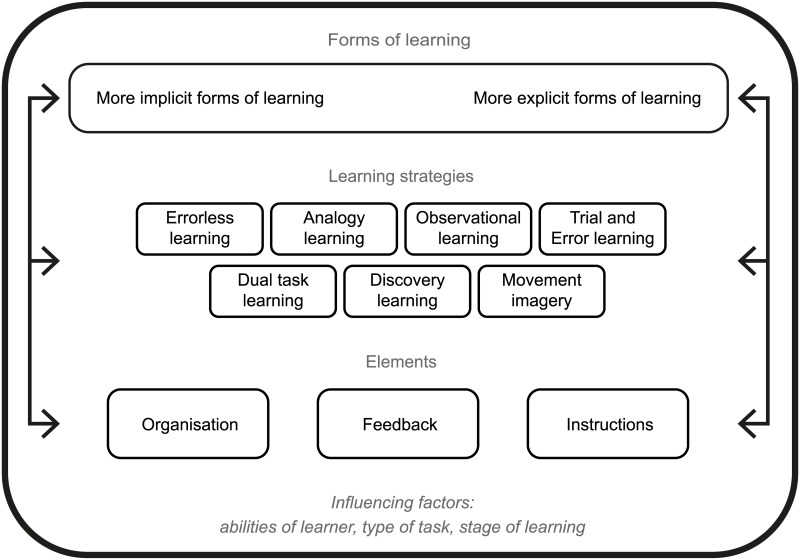
Modified figure based on suggestions of the experts.

With respect to the summary figure designed to illustrate the relationship between forms, strategies and elements, 64.7% (n = 22/34; 95% CI:46.5%-80.2%) of the experts stated that in general they agreed with the figure and 17.7% (n = 6/34; 95% CI:6.8%-34.5%) stated that they would agree if minor modifications were made. Subgroup analysis revealed no significant differences (p = 0.885) between groups. Suggestions that the experts made about the figure in the open text box could be clustered into four categories: 1) Implicit and explicit learning should be presented as a continuum and not as a dichotomy, 2) Arrows connecting implicit learning and instructions are misleading, as they suggest that implicit learning is related to instructions, 3) All three levels of the figure should be connected to each other, 4) Strategies should be arranged in a way that makes visible whether they promote more explicit or more implicit learning. Fig 3 displays the summary figure, including the following improvements: 1) Implicit and explicit learning are presented within one box to illustrate the continuum, 2) Arrows connecting implicit learning and instructions have been deleted and the position of the box ‘instructions’ was moved to the right side of the figure (side of explicit learning), 3) Arrows were adapted to connect all three levels to each other, the lines encircling the boxes were made less sharp and boxes separating the three levels were deleted to illustrate that all three levels are connected, 4) Strategies were arranged in order of whether they promote more explicit or more implicit learning. In an earlier part of the survey, the experts classified the motor learning strategies according to whether they promote more implicit or more explicit learning [16]. Arrangement of the strategies within the figure is based on this earlier classification. One of the experts who did not agree with the figure suggested that the influencing factors should be placed at the base of the figure. As the referee group agreed on that point, this suggestion was adopted.

## Discussion

The aim of the current study was to explore experts’ opinions and experiences of how motor learning can be applied in practice, using the distinction between implicit and explicit motor learning as a conceptual departure point. Answers of experts were broadly distributed. No consensus was found regarding the application of implicit and explicit motor learning. Cautious recommendations can be made on the basis of trends in the responses of the experts. Explicit motor learning can be facilitated with instructions about, for example, the goal of the task and steps and rules underlying performance. Various types of feedback (related to both performance and results of a movement) can also be used. In contrast, for implicit motor learning, instructions and feedback should be restricted. Additionally, when promoting implicit learning, an external focus of attention should be considered and the entire skill should be practiced. A reason for the variation in answers might be that, indeed, multiple options are valid depending on clinical situations. Further, answers may have been influenced by the specific practical experience of experts with respect to the target group that they worked with.

Subgroup analysis revealed no significant differences between experts from a research/theoretical field and experts from practical fields or education. This finding implies, comparable distribution of opinions between experts with a more practical role and experts with a more theoretical role.

Experts agreed that the learner’s abilities, the type of task to be learned and the stage of motor learning are the most important factors upon which to base choices when seeking to promote motor learning. Experts also seemed to agree on a summary figure that illustrated forms, strategies and elements and their relationship (64.7% of the experts agreed). It seems likely that the modified figure (Fig 3) would have achieved consensus; however, it was not presented to the expert panel. The motor learning approaches in the figure are based on strategies identified as best-known in earlier parts of the survey and should be seen as examples rather than as reflecting a comprehensive overview of all possible motor learning strategies.

The arrangement of the strategies is based on the results of an earlier survey round in which strategies were classified as being more likely to promote implicit or explicit motor learning [16]. As the results of this classification were ambiguous, the arrangement of the strategies within the figure should not be interpreted as being definitive. As suggested by the experts, most learning strategies can promote both implicit and explicit motor learning, depending on how they are shaped in practice. In the current study, many experts skipped questions regarding the application of the motor learning strategies. A reason might be that the motor learning strategies can be applied in many different ways and, depending on the application, they can promote either implicit or explicit motor learning. Therefore, the questions might have been difficult to answer without a specific context or case. Another possible explanation might be that experts knew the strategies in theory or within a research setting but did not have experiences regarding their application in practice. This finding emphasizes the importance of providing a detailed description of motor learning interventions in clinical studies, as it might not be self-evident how a motor learning strategy is applied.

Instructions, feedback and organisation are included in the figure to represent the various elements practitioners have at their disposal to shape motor learning. The list of elements used in this study is not comprehensive, but additional elements could be structured within the general terms “instructions”, “feedback” and “organisation”. For instance, “blocked practice” and “random practice” [25], may be considered as part of the organisation of practice.

The general distinction in implicit and explicit motor learning was used as a conceptual basis because many motor learning strategies and other techniques can be positioned within this distinction. We, for instance, regarded internal and external focus, as well as feedback, as options that can be applied to promote implicit or explicit motor learning. We are aware that in other studies the distinctions between internal and external focus [26–28] or the comparison of different kinds of feedback (e.g., feedback about performance versus feedback about results) [29–32] are used as a legitimate conceptual basis for motor learning.

### Strengths and Limitations

The strength of the study is that its results synthesize knowledge from a multidisciplinary, international expert panel, with published scientific and practical expertise. As suggested by recent reviews within the field of rehabilitation [33], research investigating complex interventions in practice is needed. Although this study does not evaluate the effectiveness of motor learning interventions, it may contribute to unravelling the complexity of motor learning by summarizing knowledge and experiences.

Although drawn from a variety of different backgrounds, fields and origins, the panel was not a random sample and the initial response rate with respect to participation in the survey was low, so we do not know whether their opinions are representative. Further, experts who dropped out of the study might have not have agreed with the conceptual basis, content and set-up of the study, which also might have biased the results. Therefore, all findings should be interpreted carefully and should be seen as a basis for further applied, empirical studies.

Although based on other studies and recommendations, the consensus cut-off of 70% is nevertheless arbitrary. In most Delphi studies that aim for consensus, Likert scales are used (e.g., [21,22,34]). We decided not to use this kind of response option as it probably would have hindered the exploratory character of the study. When using Likert scales, sufficient support from literature (or other sources) is needed for the statements to be rated. However, in the case of motor learning, the literature varies and sometimes even is contradictory. Therefore, response options that allowed participants to signal different views were used.

The purpose of the presented figure was to provide an overview of possible ways to apply motor learning, and their relationships, when using the implicit-explicit distinction as a conceptual basis. Therefore, the figure might support communication about motor learning. In recent years, numerous figures, models and programs based on different conceptual backgrounds have been developed to explain motor learning in different target groups (e.g., [35–38]). The advantages of the current figure, however, are that it has been developed with input from international experts from different fields, and it signals both options for motor learning and factors that should be taken into account when choosing motor learning content.

### Implications for practice and future research

Results of the current study may help practitioners (e.g., physical therapists), especially those who are less experienced, by providing options and examples of theoretically underpinned methods of facilitating motor learning. Perhaps more importantly, students and neophyte professionals need to understand that there are factors worthy of consideration when preparing and conducting a motor learning session. Future research should focus on evaluation and comparison of the effects of different applications, taking into account the influencing factors identified in this study. It would also be of interest to investigate how practitioners currently make choices at a case level when promoting motor learning and to assess the value of the presented summary figure for decision-making in daily practice.

## Supporting Information

S1 Fig Recruitment and compilation of experts. (PDF)

S1 Table Questions asked within the survey. (PDF)
