# Supplementary material for: Multidisciplinary Views on Applying Explicit and Implicit Motor Learning in Practice: An International Survey
Source: PLoS One. 2015 Aug 21;10(8):e0135522. doi: 10.1371/journal.pone.0135522 (PMC4546413; doi:10.1371/journal.pone.0135522)
Supplement: S1 Fig — (PDF) [file pone.0135522.s001.pdf]

Supporting Information 1: Recruitment and compilation of experts.

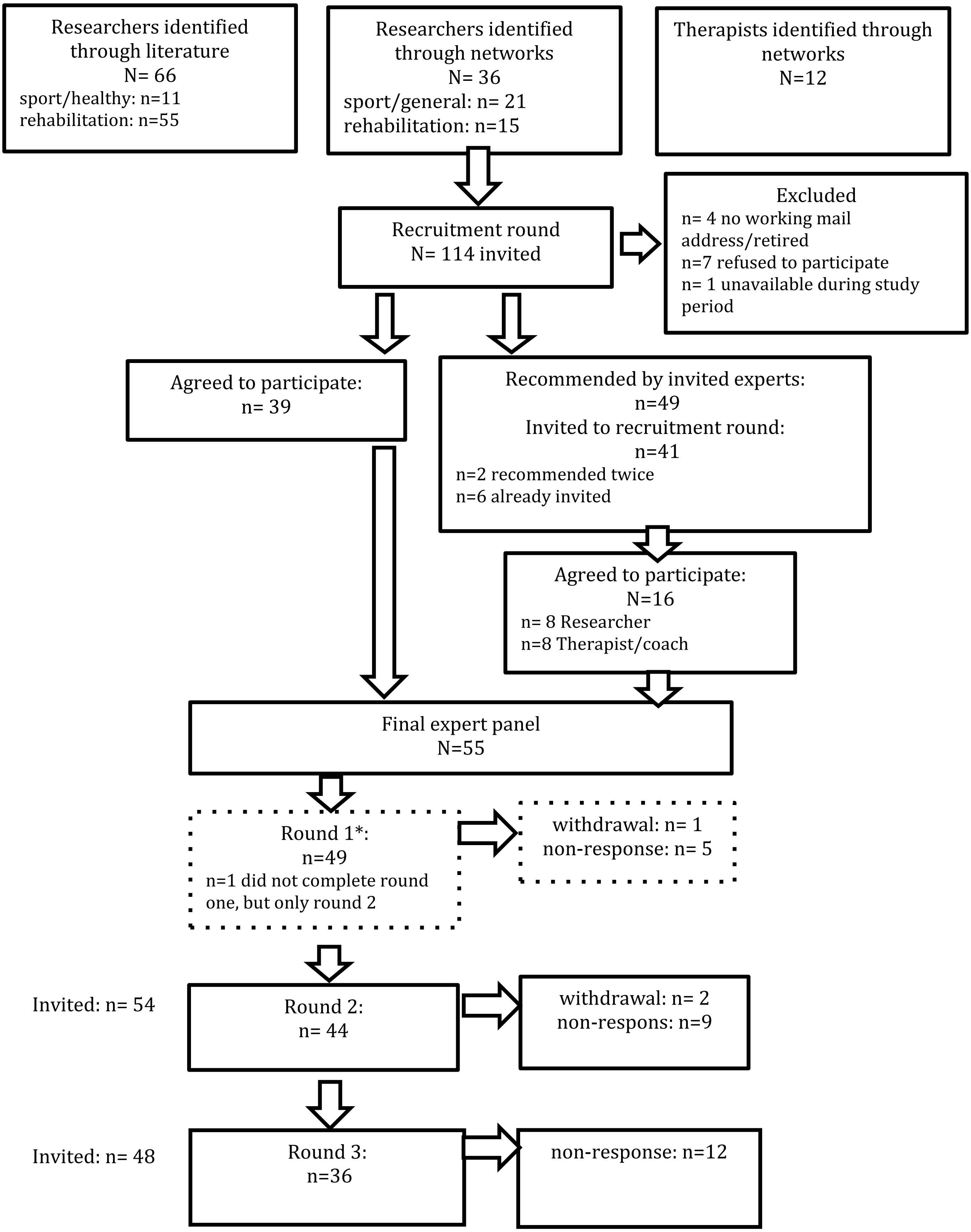

**Boxes of Round 1 are dashed as results of this round are not taken into account in this article*
