# Supplementary material for: Multidisciplinary Views on Applying Explicit and Implicit Motor Learning in Practice: An International Survey
Source: PLoS One. 2015 Aug 21;10(8):e0135522. doi: 10.1371/journal.pone.0135522 (PMC4546413; doi:10.1371/journal.pone.0135522)
Supplement: S1 Table — (PDF) [file pone.0135522.s002.pdf]

Supporting Information 2: Questions asked within the survey

Element	Question	Answering method	Answering options
**Instructions**	Should any specific instructions be given to the learner about the task?	Open comment box	-
**Focus of attention**	Should the learner be instructed to focus on:	Multiple choice; one option	• Internal cues (internal focus of attention)<br>• External cues (external focus of attention)<br>• Not applicable<br>• I cannot state as it depends on (open comment possible)
**Manual guidance**	Should manual guidance be used?	Multiple choice; one option	• No<br>• Little<br>• Some<br>• Much<br>• Not applicable<br>• I cannot state as it depends on (open comment possible)
**Feedback (content)**	Which forms of feedback should the learner receive?	Multiple choice; more options	• Feedback on the performance<br>• Feedback on the results<br>• Addressing the aspects of the performed skill which are good;<br>• Addressing the aspects of the performed skill which should be improved<br>• Not applicable<br>• I cannot state as it depends on (open comment possible)
**Feedback (timing)**	When should the feedback be given?	Multiple choice; more options	• During the movement<br>• After the movement<br>• Immediately after the relevant action<br>• Delayed after the relevant action

Note: Questions were asked separately for implicit and explicit motor learning
